# ZMIZ2 promotes the development of triple-receptor negative breast cancer

**DOI:** 10.1186/s12935-021-02393-x

**Published:** 2022-01-31

**Authors:** Xiaopan Zou, Yan Liu, Jun Di, Wei Wei, Nobumoto Watanabe, Jiang Li, Xiaomeng Li

**Affiliations:** 1 The Key Laboratory of Molecular Epigenetic, Institute of Genetics and Cytology, Northeast Normal University, No.5268 Renmin Street, Nanguan District, Changchun, 130024 Jilin China; 2 Breast and Thyroid Surgery, Jilin Province People’s Hospital, Changchun, 130021 Jilin China; 3 Pathological Diagnostic Center, Jilin Province People’s Hospital, Changchun, 130021 Jilin China; 4 Bio-Active Compounds Discovery Research Unit, RIKEN Center for Sustainable Resource Science, Wako, Saitama Japan; 5 Affiliated Stomatology Hospital of Guangzhou Medical University, Guangzhou, 510180 Guangdong China

**Keywords:** Triple-receptor negative breast cancer, ZMIZ2, Survival analysis, Regulatory network

## Abstract

**Background:**

Triple-receptor negative breast cancer (TNBC) is an aggressive breast tumor subtype that generally has a poor prognosis. This study aimed to investigate the role and regulatory mechanisms of Zinc finger MIZ-type containing 2 (ZMIZ2) in relation to TNBC.

**Methods:**

Based on data from The Cancer Genome Atlas (TCGA), the expression of ZMIZ2 in different subtypes and its correlation with androgen receptor (AR) were analyzed, and a regulatory mechanism network was constructed. The expression and prognostic value of ZMIZ2 in clinical TNBC tissue samples were also investigated. Furthermore, in vitro studies were conducted to investigate the effects of ZMIZ2 knockdown on the malignant behaviors of TNBC cells and target gene expression.

**Results:**

Based on TCGA data, ZMIZ2 was found to be significantly upregulated in TNBC tissues and its expression was negatively correlated with AR expression. Key relationships, such as the ZMIZ2-CCL5, ZMIZ2/AR-MCM3, ZMIZ2/AR-E2F4, and the ZMIZ2/AR-DHX38 were identified, which were enriched in NOD-like receptor signaling pathway/toll-like receptor signaling pathway, DNA replication, cell cycle, and spliceosome, respectively. Moreover, ZMIZ2 was upregulated in clinical breast cancer tissues and its high expression was correlated with the poor prognosis of TNBC patients. Furthermore, ZMIZ2 expression was increased in breast cancer cells, and a knockdown of ZMIZ2 inhibited MDA-MB-231 cell proliferation, migration, and invasion, induced cell cycle arrest in the G1 phase, and promoted cell apoptosis. Furthermore, ZMIZ2 knockdown inhibited the mRNA and protein expression of CCL5, MCM3, E2F4, and DHX38.

**Conclusion:**

Our findings reveal that ZMIZ2 is upregulated in TNBC tissues and is associated with its poor prognosis. ZMIZ2 may promote TNBC progression by promoting the expression of its target genes and affecting the corresponding pathways. Consequently, ZMIZ2 may serve as a promising target for future TNBC treatments.

**Supplementary Information:**

The online version contains supplementary material available at 10.1186/s12935-021-02393-x.

## Background

Breast cancer is the most common malignancy among women worldwide [1]. Among the different types of breast cancers, tumors that are immunohistochemically characterized as estrogen receptor (ER)-negative, progesterone receptor (PR)-negative, and human epidermal growth factor receptor 2 (HER2)-negative are classified as triple-receptor negative breast cancers (TNBCs), and they account for 15–20% of all breast cancers [2]. Compared with HER2-positive or hormone receptor-positive breast cancers, TNBCs have a more aggressive clinical course, earlier relapses, and a poorer overall prognosis, and thus represent a major challenge for patients and physicians [3, 4]. As the molecular mechanisms driving TNBC progression have not yet been fully elucidated, targeted therapies have not yet significantly improved overall survival in TNBC patients [5]. Therefore, the development of novel targets for TNBC represents a critical and yet unmet clinical need.

Zinc finger MIZ-type containing 2 (ZMIZ2), also named ZIMP7, is a protein inhibitor of activated STAT (PIAS)-like protein and a transcriptional coactivator [6]. ZMIZ2 contains a C-terminal transactivation domain that can augment androgen receptor (AR)-mediated transcription [7, 8]. AR has been suggested to play a critical role in male sexual development, as well as in the growth and survival of normal and malignant prostate cells [6]. ZMIZ2 is highly expressed in the testis and promotes the growth of prostate cancer cells [6]. However, the role of ZMIZ2 in TNBC has not, to the best of our knowledge, been widely reported. AR seems to play an important role in the carcinogenesis of TNBC, as it is reportedly expressed in 10–50% of TNBC cases [9], and those with an the AR-positive subtype are susceptible to AR blockades [9–11]. Given the regulatory relationship between ZMIZ2 and AR, as well as the role of AR in TNBC, we hypothesize that ZMIZ2 plays a key role in the carcinogenesis of TNBC, and thus, may serve as a novel biomarker for TNBC.

To confirm our speculations, we downloaded RNA-seq data and clinical information for breast cancer from The Cancer Genome Atlas (TCGA) database and analyzed it for ZMIZ2 expression and the correlation between ZMIZ2 and AR. The potential regulatory mechanisms of ZMIZ2 in TNBC were also explored using comprehensive bioinformatics analyses, such as differential expression analysis, protein–protein interaction analysis, pathway analysis and regulatory mechanism network construction. Moreover, the expression and prognostic value of ZMIZ2 in clinical TNBC tissue samples were investigated. Furthermore, in vitro studies were conducted to determine the expression, function, and mechanisms of ZMIZ2 in the TNBC cells. These findings will help to facilitate the development of novel therapeutic strategies for TNBC in the future.

## Methods

### Bioinformatics analysis of ZMIZ2 expression in different breast cancer subtypes and its regulatory mechanisms in TNBC using TCGA data

#### Acquisition of RNA-seq data and clinical information

The RNA-seq level 3 data and clinical information for breast cancer were downloaded from TCGA database (https://cancergenome.nih.gov/). A total of 845 samples were divided into luminal (580), HER2 (30), and TNBC (122) subtypes based on ER, PR, and HER2 status, and there were 113 normal samples. Additionally, according to their HER2 status, the samples were divided into HER2+ (110) and HER2− (622) groups.

#### Analysis of ZMIZ2 expression in different breast cancer subtypes and its co-expression with AR

To explore the role of ZMIZ2 in the different breast cancer subtypes, data about its expression was obtained from TCGA. Moreover, the correlation between ZMIZ2 and AR in the luminal, HER2, TNBC, HER2+, HER2−, and normal samples was separately calculated using the Pearson algorithm [12]. When p < 0.05, the relationship was considered to be a co-expression.

#### Differential expression analysis

Based on the RNA-seq level 3 data obtained from TCGA database, the differentially expressed (DE) mRNAs in TNBC when compared with the normal samples were calculated using the limma packet [13] of R language. The thresholds were set as |log fold change (FC)|> 0.75 and adj. p value < 0.05. Moreover, the differentially expressed (DE) mRNAs in TNBC when compared with the normal samples and with the HER2 samples, were identified using threshold values of |logFC|> 0.5 and adj. p value < 0.05.

#### PPI and transcriptional regulatory analyses

Based on the information of the STRING website [14], the PPIs for all the DE mRNA-corresponding proteins were predicted. We extracted the PPI for ZMIZ2 with the other DE mRNA-corresponding proteins, and constructed PPI networks.

Based on the transcription factor (TF)-target relations in the ENCODE database [15], a TF that could regulate ZMIZ2 was predicted from the DE mRNAs.

#### Gene set enrichment analysis (GSEA) of ZMIZ2 regulatory pathways and construction of its regulatory mechanism network

GSEA [16] was performed for TNBC, HER2, and normal samples. Based on the GSEA method, the positive and negative pathways of ZMIZ2 in 122 TNBC samples were identified using metrics to rank the genes using Pearson with ZMIZ2 as the key for the phenotypic labels. The thresholds were set to |NES|> 1.5 and p < 0.05. Using the same methods, the pathways involving ZMIZ2, in 113 normal and 30 HER2 samples, were analyzed. Additionally, Venn analyses of the positive and negative pathways between the TNBC and normal samples, as well as between the TNBC and HER2 samples, were performed. Based on results of the GSEA, we further constructed regulatory mechanism networks for ZMIZ2 based on the pathways common between the TNBC and normal samples, and TNBC and HER2 samples. Due to the large initial network, we screened genes that had r correlation values > 0.3 with ZMIZ2 to use for the construction of the network.

Considering the correlation between ZMIZ2 and AR, GSEA for AR in 122 of the TNBC samples was also performed to analyze its positive and negative pathways, and a Venn analysis of this data was conducted. Based on the GSEA results, the genes that were significantly correlated with ZMIZ2 and AR (|r|> 0.3) and enriched in four pathways related to proliferation regulation (including spliceosome, DNA replication, cell cycle, and homologous recombination) were selected to construct the regulatory mechanism network for ZMIZ2-AR.

### Clinical analysis of ZMIZ2 expression in different breast cancer subtypes and the prognostic value of ZMIZ2 for TNBC when using clinical samples

#### Tissue sample collection

Breast cancer tissue samples (21 luminal, 21 HER2, and 24 TNBC) and 25 normal breast tissue samples were collected during 2018–2021. Each sample was divided into three parts for real-time quantitative reverse transcription-PCR (qRT-PCR), western blot, and immunohistochemical assays. The tissue samples for RNA and protein extraction were stored in liquid nitrogen within 2 weeks. The tissue resections for the immunohistochemical assay were fixed in formalin and embedded in paraffin. In addition to these samples, we retrospectively collected the paraffin specimens of patients with TNBC during 2008–2018 and assessed their ZMIZ2 expression. Based on the ZMIZ2 expression in all samples and follow-up information, survival analysis was conducted. This study was approved by the Ethics Committee of Jilin Province People’s Hospital. Informed consent was signed by all participants.

#### qRT-PCR

Total RNA was extracted using an RNA extraction solution (Servicebio, Wuhan, China). Reverse transcription was performed using a Servicebio^®^RT First Strand cDNA Synthesis kit (Servicebio), followed by PCR reactions with 2 × SYBR Green qPCR Master Mix (Servicebio). GAPDH was used as an internal control for mRNA. The 2^−ΔΔCT^ method was used for relative quantification. The primers (5′–3′) used for gene amplification are listed in Table 1.

**Table 1:** The primers (5′–3′) used in this study

Primers	Forward	Reverse
ZMIZ2	CCTGGCTGTAAGCAACCATGTC	GCCAGTTGGTGTTCATCTGCCG
MCM3	CGAGACCTAGAAAATGGCAGCC	GCAGTGCAAAGCACATACCGCA
CCL5	CCTGCTGCTTTGCCTACATTGC	ACACACTTGGCGGTTCTTTCGG
E2F4	GGAAGGTATCGGGCTAATCGAG	AGCTCCTCGATCTCTGCCTTGA
DHX38	GACCTGGATCACTACAGTGCCA	GTGGCTGATGTGACGATGAGCT
GAPDH	GGAGCGAGATCCCTCCAAAAT	GGCTGTTGTCATACTTCTCATGG

#### Western blot assay

The samples were washed and lysed with RIPA buffer (Servicebio). Total proteins were extracted using a protein extraction kit (Nanjing KeyGen Biotech Co., Ltd., Nanjing, China). Before being transferred to the polyvinylidene fluoride (PVDF) membranes (Millipore, USA), total proteins (30–50 μg/per lane) were first separated on 10% sodium dodecyl sulfonate-polyacrylamide gels. The membranes were blocked with 5% skim milk for 1 h, and then the primary antibodies [anti- ZMIZ2 (Abclonal, Wuhan, China), anti-CCL5 (Bioss, Beijing, China), anti-MCM3 (Abclonal), anti-E2F4 (Bioss), and anti-DHX38 (Bioss)] were added and incubated at 4 °C overnight. Following this, the membranes were conjugated with a second antibody (Bioss) for 30 min at 37 °C. Protein bands were visualized using an ECL detection kit (Servicebio). The intensities of the target bands were quantified by densitometry using AlphaEaseFC Software (Alpha Innotech, USA).

#### Immunohistochemistry

The paraffin sections (4-μm-thick) were soaked in 3% H_2_O_2_ for 10 min after dewaxing with xylene and alcohol baths. The sections were then soaked in antigen retrieval solution for 5 min. Then, PBS with 10% sheep serum (Gibco, Grand Island, NY, USA) was added to the section to block the non-specific sites. The primary antibody against ZMIZ2 (Abclonal) was added and then incubated at 4 °C overnight, followed by the incubation with the corresponding secondary antibody (Bioss). The images (200 × magnification) were photographed and the positive area density was analyzed using an Image-Pro Plus 6.0 System (Media Cybernetics Corporation, USA).

#### Survival analysis

To better understand the prognostic value of ZMIZ2 and MCM3 in TNBC, survival analysis was conducted. The TNBC samples were divided into high- and low-expression groups based on the median values of their gene expression. Kaplan–Meier survival curves were drawn using the R package based on the overall survival information (OS status and OS time) of the TNBC samples. Log-rank statistical tests were then performed, and a p < 0.05 indicated statistical significance.

### In vitro studies of ZMIZ2 expression in different breast cancer cell lines and the function and mechanisms of ZMIZ2 in TNBC cells

#### Cell culture

The breast epithelial cell line MCF-10A (American Type Culture Collection (ATCC), Manassas, VA) was maintained in Dulbecco’s Modified Eagle’s Medium (DMEM)/Ham’s F12 Medium (Gibco) supplemented with 20 ng/mL epidermal growth factor, 5% horse serum, and 500 ng/mL hydrocortisone (Life Technologies, USA). The luminal subtype cell line MCF-7, HER2 subtype cell line SKBR-3, and TNBC cell line MDA-MB-231 (ATCC) were cultured in DMEM supplemented with 10% fetal bovine serum (FBS), penicillin, and streptomycin (Life Technologies, USA). All cells were cultured in a 5% CO_2_ cell incubator at 37 °C. Notably, MDA-MB-231 cell line was authenticated by Short Tandem Repeat (STR) profiling before initiation of this study. ZMIZ2 mRNA and protein level expression levels were determined via qRT-PCR and western blot assays.

#### Cell transfection

Short hairpin RNAs (shRNAs) targeting ZMIZ2 were designed and their sequences are shown in Additional file 2: Table S1. They were cloned into the plenti-shRNA-GFP-puro vector (Shanghai GenePharma Co., Ltd.). The shRNA nontarget sequence was cloned into the lentiviral vector as a negative control. Positive colonies were selected by sequencing. The plenti-shRNA-GFP-puro recombinant vector and lentiviral packaging mix were subsequently transfected into HEK-293T cells using Effectene Transfection Reagent (QIAGEN, Germantown, MD). After 48 h of incubation, the supernatant containing recombinant lentivirus was harvested to assess transfection efficiency by detecting ZMIZ2 expression with qRT-PCR. Moreover, MDA-MB-231 cells were also transfected with plenti-shRNA-GFP-puro recombinant vector using the same Effectene Transfection Reagent to confirm the transfection efficiency.

#### CCK8 assay

MDA-MB-231 cells were seeded onto 96-well plates. Cell viability was evaluated using the CCK-8 kit (cat#E606335-0500, BBI Life Sciences, New York, USA) in accordance with the manufacturers’ instructions. Briefly, 10 μL of CCK8 solution was added to the cells, followed by incubation for 1 h at 37 ℃. The absorbance at 450 nm was then measured using a microplate reader (Epoch2, BioTek, USA).

#### Detection of cell apoptosis

Cell apoptosis was detected using the Annexin V-FITC/PI Apoptosis Detection kit (cat#40302ES20; Yeasen, Pudong, Shanghai, China). Briefly, MDA-MB-231 cells in different transfection groups were digested with trypsin without EDTA and then collected by centrifugation. The cells were washed with pre-chilled PBS and then resuspended in 1 × binding buffer with a density of 1 × 10^6^ cells/mL. Subsequently, 100 μL cell suspension was stained with 5 μL Annexin V-FITC and 10 μL propidium iodide (PI) for 15 min at room temperature away from the light. Cell apoptosis was then detected using a flow cytometer (FC500, Beckman Coulter, Fullerton, CA, USA).

#### Transwell assays

Cell migration and invasion were assessed using Transwell assays. Briefly, MDA-MB-231 cells in different transfection groups were suspended in serum-free medium, and 100 μL of cell suspension was then seeded into the upper chamber of the Transwell insert (Corning, Costar, Cambridge, MA, USA). Notably, the insert was coated with Matrigel for the invasion assays. The lower chamber of the Transwell insert was filled with 600 μL of medium containing 20% FBS as a chemoattractant. After 24 h of incubation, the Transwell insert was washed with PBS, fixed with formaldehyde (BBI Life Sciences) for 30 min, and then stained with 0.1% crystal violet (BBI Life Sciences) for 30 min. The non-migrated or non-invaded cells remaining on the upper chamber were removed with cotton wool. Then, the cells that had migrated or invaded the membrane in three randomly selected fields (100 × magnification) were observed and counted using an inverted fluorescence microscope (CKX41, Olympus, Tokyo, Japan).

#### Cell cycle analysis

To estimate the dissemination of the MDA-MB-231 cells in the different phases of the cell cycle, MDA-MB-231 cells in different transfection groups were digested with trypsin and fixed with pre-cool 70% ethanol overnight at 4 °C. Cells were collected by centrifugation, suspended with PBS, and then incubated with 2.5 μL of RNase (10 mg/mL) for 30 min at 37 °C. The cells were then stained with 50 μL PI (10×, cat#E607306; Sangon Biotech Co., Ltd., Shanghai, China). Cell distribution was then determined within 2 h using a flow cytometer (FC500, Beckman Coulter).

#### Validation of mRNA and the protein expression of the key targets of ZMIZ2

Based on the GSEA results, the key prognostic genes in the regulatory mechanism networks of ZMIZ2 were selected for validation as they were correlated with ZMIZ2 and had prognostic value in TNBC. Four targets of ZMIZ2 were selected, including the C–C Motif chemokine ligand 5 (CCL5), minichromosome maintenance complex component 3 (MCM3), E2F transcription factor 4 (E2F4), and DEAH-box helicase 38 (DHX38). The mRNA and protein expression of these targets was quantified via qRT-PCR and western blot assays.

### Statistical analysis

GraphPad Prism v6.0 (GraphPad V6.0 Software Inc., San Diego, CA, USA) was used for data analysis. The data are shown as mean ± standard deviation (SD), followed by normal distribution tests. If the measurement data conformed to a normal distribution, a Student’s t-test was used for comparison to assess significant differences between two groups. If not, significant differences were analyzed using a Mann–Whitney U test. The Kaplan–Meier survival analysis was conducted, followed by a Log-rank statistical test. P < 0.05 was considered statistically significant.

## Results

### Bioinformatics analysis revealed the increased expression of ZMIZ2 expression in breast cancers and its regulatory mechanisms in TNBC using TCGA data

#### Expression of ZMIZ2 between different breast cancer subtypes and its correlation with AR

Based on TCGA data, the differences in ZMIZ2 expression between the different subtypes were analyzed. The expression of ZMIZ2 was significantly up-regulated in TNBC samples when compared to normal samples (p = 2.44E−32) or HER2 samples (p = 1.54E−07; Table 2). Moreover, the correlation between ZMIZ2 and AR in different subtypes of breast cancers was calculated (Table 3). We found negative correlations between ZMIZ2 and AR in the cancer tissue samples (r < 0), while the correlation was positive in the normal tissue samples (r > 0). Additionally, the correlations were significant in the TNBC, luminal, HER2−, and all breast cancers samples, but there was no significant correlation in HER2 and HER2 + samples.

**Table 2:** The difference of *ZMIZ2* expression between different subtypes

Group	logFC	P value
Cancer-Normal	0.436174019	9.88E−17
TNBC-Normal	0.786992	2.44E−32
HER2-Normal	0.260674	0.00946
Lum-Normal	0.371459	3.23E−13
Lum-TNBC	0.415533	5.76E−17
Lum-HER2	0.110785	0.225652
TNBC-HER2	0.526317	1.54E−07
HER2 (POS-NEG)	− 0.19074	0.000279

**Table 3:** The correlations between* ZMIZ2* and *AR* in different subtypes of breast cancers as well as all breast cancer and normal tissues

Breast cancer subtypes	Gene 1	Gene 2	r	P value
All breast cancers	ZMIZ2	AR	− 0.32766	< 2.2e−16
TNBC	ZMIZ2	AR	− 0.37441	2.15E−05
LUM	ZMIZ2	AR	− 0.13049	0.001636
HER2	ZMIZ2	AR	− 0.06272	0.742
HER2+	ZMIZ2	AR	− 0.04444	0.6387
HER2-	ZMIZ2	AR	− 0.3448	< 2.2e−16
Normal	ZMIZ1	AR	0.4078161	7.344e−06

#### Identification of DE mRNAs

**Fig. 1:**
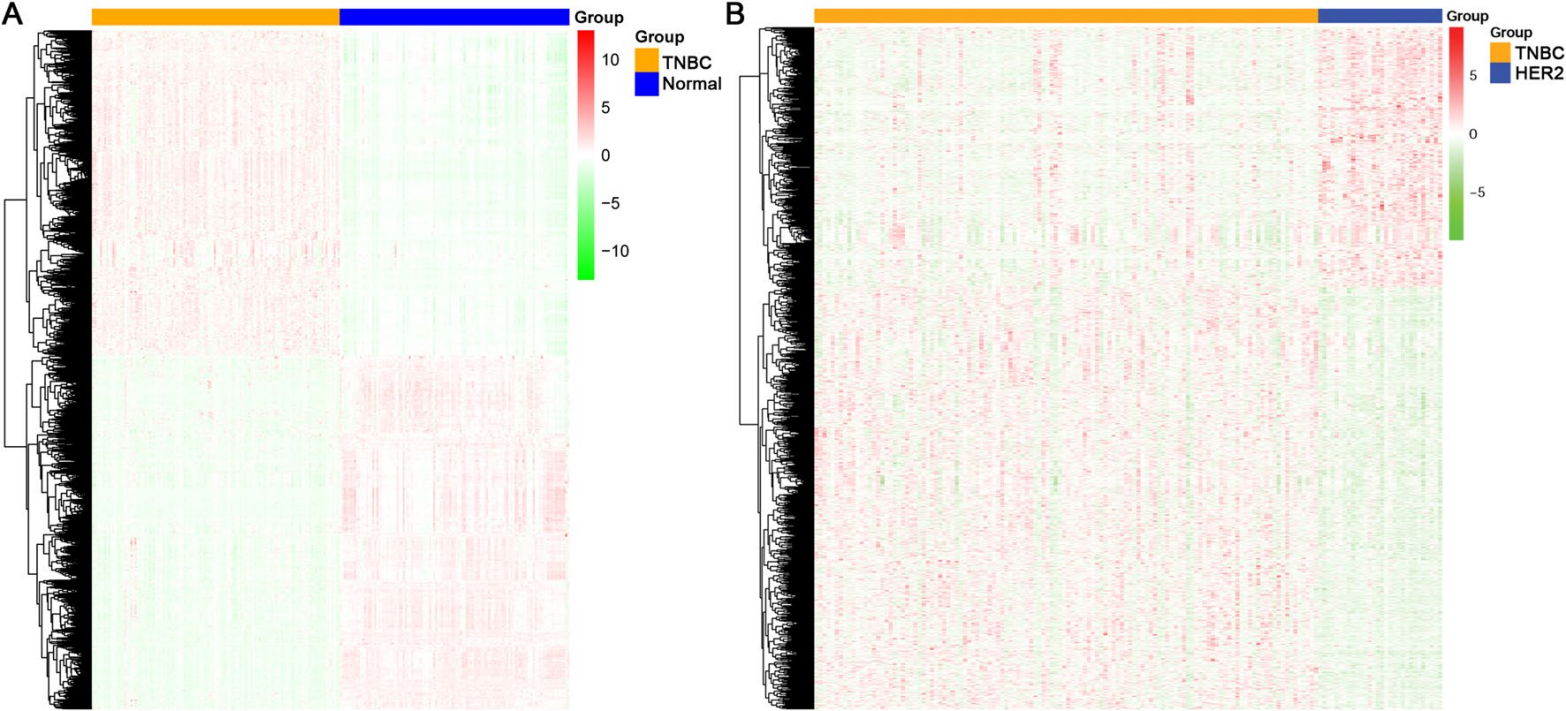
A hierarchical clustering heatmap of the differentially expressed mRNAs (DE mRNAs). **A** Heatmap of the DE mRNAs between TNBC and normal samples. **B** Heatmap of the DE mRNAs between the TNBC and HER2 samples

Based on the thresholds of |logFC|> 0.75 and adj.p value < 0.05, 3833 mRNAs differentially expressed between the TNBC and normal samples were selected, 1729 and 2104 of which were upregulated and downregulated, respectively. The hierarchical clustering heatmap is shown in Fig. 1A. The TNBC and normal samples were well distinguished by these DE mRNAs. Moreover, a total of 2012 DE mRNAs (1240 upregulated and 772 downregulated) between the TNBC and HER2 samples were identified with the thresholds of |logFC|> 0.5 and adj.p value < 0.05 (Fig. 1B).

#### PPI networks and transcriptional regulatory relationships between ZMIZ2 and DE mRNAs

**Fig. 2:**
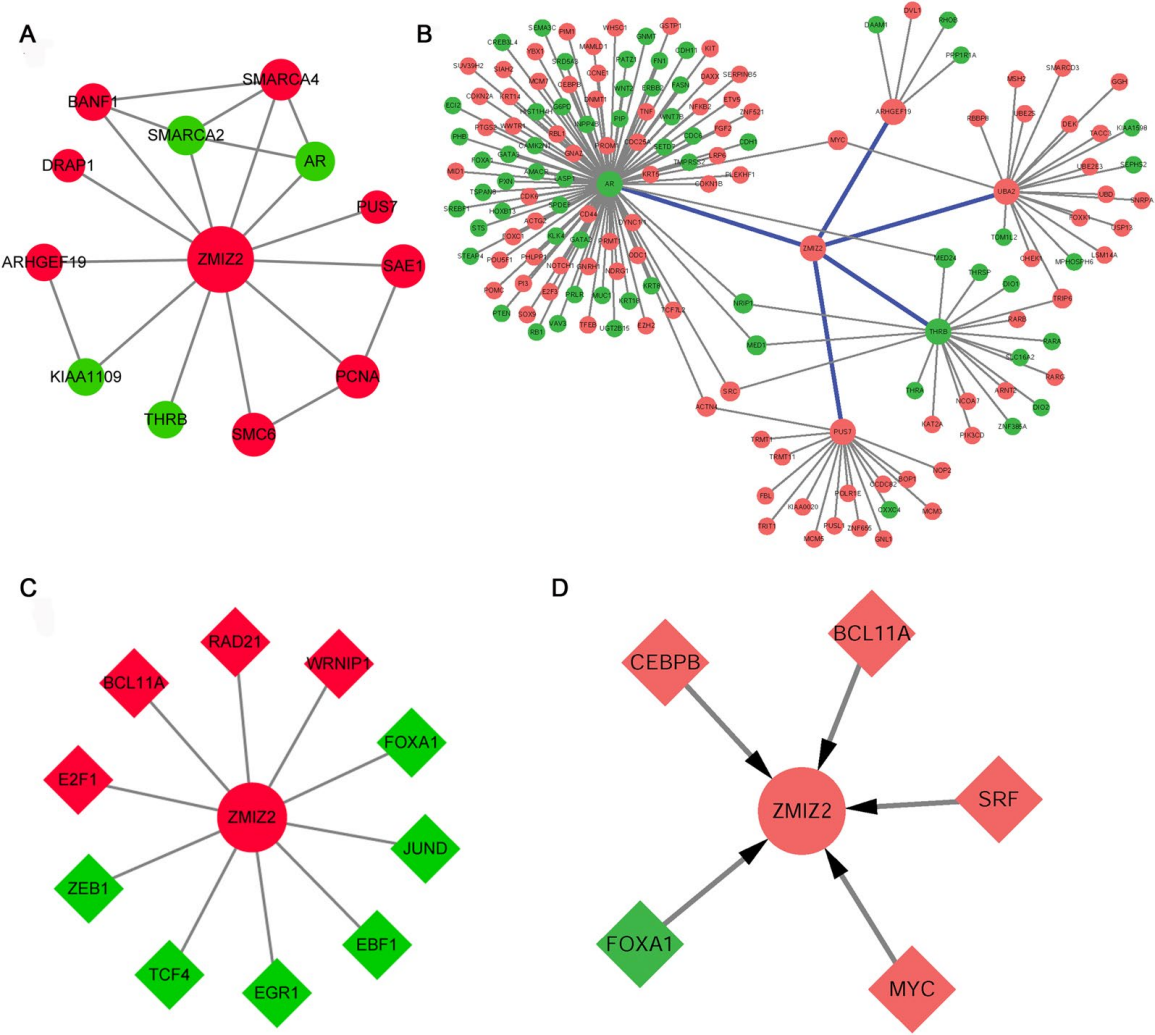
ZMIZ2-related protein–protein interaction (PPI) networks and transcription factor regulatory networks. **A** PPI networks constructed for ZMIZ2 and the DE mRNAs between TNBC and normal samples. **B** PPI networks constructed for ZMIZ2 and the DE mRNAs between the TNBC and HER2 samples. **C** Transcription factor regulatory networks constructed for ZMIZ2 and the DE mRNAs between the TNBC and normal samples. **D** Transcription factor regulatory networks constructed for ZMIZ2 and the DE mRNAs between the TNBC and HER2 samples. Red circle nodes represent upregulated genes and green circle nodes represent downregulated genes. Red rhombus nodes represent upregulated transcription factors, and green rhombus nodes represent downregulated transcription factors

Based on the information from the STRING database, the PPI pairs of DE mRNAs between the TNBC and normal samples were analyzed, and 3833 nodes and 44832 interaction pairs were identified. The PPIs between ZMIZ2 and these DE mRNA-corresponding proteins were then predicted, and the PPI network was constructed using 12 DE mRNAs (SMARCA4, BANF1, DRAP1, ARHGEF19, KIAA1109, THRB, SMC6, PCNA, SAE1, PUS7, AR, and SMARCA2) and ZMIZ2 (Fig. 2A). Moreover, the PPI pairs of the DE mRNAs among the TNBC and HER2 samples were predicted, and 852 nodes and 3688 PPI pairs were identified. Then, five DE mRNAs interacting directly with ZMIZ2 were screened, and the DE mRNAs that could interact directly with them were further identified to construct the PPI network (Fig. 2B). Notably, an interaction between ZMIZ2 and AR was observed in the two PPI networks mentioned above (Fig. 2A, B).

The transcriptional regulatory relationship between ZMIZ2 and the DE mRNAs in TNBC vs. the normal group was predicted, and 10 DE mRNAs (WRNIP1, RAD21, BCL11A, E2F1, ZEB1, TCF4, EGR1, EBF1, JUND, and FOXA1) were found to be TFs of ZMIZ2 (Fig. 2C). Furthermore, the transcriptional regulatory relationships between ZMIZ2 and DE mRNAs belonging to the TNBC and HER2 groups were predicted, and 5 DE mRNAs (CEBPB, BCL11A, MUC, SRF, and FOXA1) were found to be TFs of ZMIZ2 (Fig. 2D).

#### The ZMIZ2 regulatory pathway in TNBC, HER2, and normal samples

Using ZMIZ2 as phenotype labels, the positive and negative pathways (top 20 based on NES) regulated by ZMIZ2 in the TNBC, normal, and HER2 samples are shown in Additional file 2: Tables S2–S4.

**Fig. 3:**
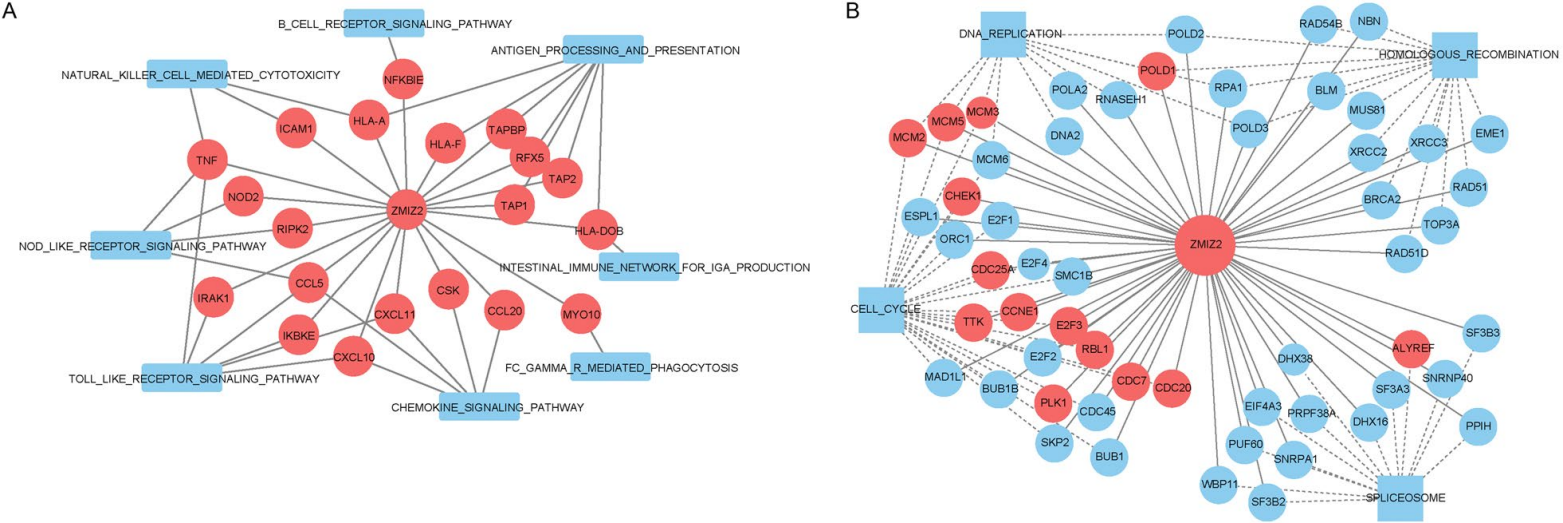
Gene-pathway regulatory mechanism networks for ZMIZ2 based on the gene set enrichment analysis (GSEA) results. **A** Regulatory mechanism networks of ZMIZ2 based on the pathways between the TNBC and normal samples. **B** Regulatory mechanism networks of the ZMIZ2 based on the pathways between the TNBC and HER2 samples. Red circle nodes represent upregulated genes, and blue circle nodes represent non-differentially expressed genes. Square nodes represent pathways

Venn analysis showed the key pathways regulated by ZMIZ2, and there were obvious differences between the TNBC and normal samples (Additional file 1: Fig. S1A). Among the top 20 (based on NES) pathways in the TNBC-positive group, were 8 immune system-related and 6 immune disease-related pathways (Table 4). We focused on the immune-related pathways in the following analyses. Based on the analysis results of GSEA, we further constructed the regulatory mechanism network of ZMIZ2. Due to the large initial network, we selected the DE mRNAs that had r correlations > 0.3 with ZMIZ2 to construct the network. Finally, a network with 29 nodes (8 pathway nodes and 21 gene nodes) and 48 edges was constructed (Fig. 3A). In this network, CCL5, which had higher node degrees, was significantly enriched in toll-like receptor signaling pathways, NOD-like receptor signaling pathways, and chemokine signaling pathways.

**Table 4:** Among the top 20 (based on NES) pathways in TNBC-positive group, there were 8 immune system-related and 6 immune disease-related pathway

Name	NES	Pathway type
KEGG_ALLOGRAFT_REJECTION	2.5076501	Immune disease
KEGG_LEISHMANIA_INFECTION	2.4929893	Infectious disease: parasitic
KEGG_NOD_LIKE_RECEPTOR_SIGNALING_PATHWAY	2.416907	Immune system
KEGG_GRAFT_VERSUS_HOST_DISEASE	2.3912017	Immune disease
KEGG_ANTIGEN_PROCESSING_AND_PRESENTATION	2.3886569	Immune system
KEGG_PRIMARY_IMMUNODEFICIENCY	2.3744943	Immune disease
KEGG_NATURAL_KILLER_CELL_MEDIATED_CYTOTOXICITY	2.350802	Immune system
KEGG_TYPE_I_DIABETES_MELLITUS	2.3472748	Endocrine and metabolic disease
KEGG_CHEMOKINE_SIGNALING_PATHWAY	2.3357153	Immune system
KEGG_SYSTEMIC_LUPUS_ERYTHEMATOSUS	2.3204288	Immune disease
KEGG_B_CELL_RECEPTOR_SIGNALING_PATHWAY	2.2692728	Immune system
KEGG_INTESTINAL_IMMUNE_NETWORK_FOR_IGA_PRODUCTION	2.247273	Immune system
KEGG_AUTOIMMUNE_THYROID_DISEASE	2.2273605	Immune disease
KEGG_TOLL_LIKE_RECEPTOR_SIGNALING_PATHWAY	2.2212996	Immune system
KEGG_FC_GAMMA_R_MEDIATED_PHAGOCYTOSIS	2.1360247	Immune system
KEGG_ASTHMA	2.0703006	Immune disease
KEGG_PROTEASOME	2.0634766	Folding, sorting and degradation
KEGG_VIRAL_MYOCARDITIS	1.9749774	Cardiovascular disease
KEGG_BASAL_TRANSCRIPTION_FACTORS	1.934293	Transcription

Venn analysis showed the key pathways regulated by ZMIZ2, and there were obvious differences between the TNBC and HER2 samples (Additional file 1: Fig. S1B). Among the positive pathways regulated by ZMIZ2, 26 common pathways were obtained in both TNBC and HER2 samples, such as T cell receptor signaling pathway, allograft rejection, and NOD like receptor signaling pathway, most of which were immune-related pathways. These data suggested that both the TNBC and HER2 subtypes were closely associated with immunity. In addition, among the positive pathways regulated by ZMIZ2, 51 were TNBC-specific, most of which were associated with proliferation, such as the spliceosome, DNA replication, cell cycle, and homologous recombination. These data revealed that ZMIZ2 may be involved in TNBC by regulating the pathways related to proliferation. Based on the analysis results of GSEA, we further constructed the regulatory mechanism network of ZMIZ2. Due to the large initial network, we selected the genes that had r correlations > 0.3 with ZMIZ2 to construct the network. Finally, a network with 60 nodes (4 pathway nodes and 56 gene nodes) and 118 edges was constructed (Fig. 3B). Key relationships, such as ZMIZ2-MCM3, ZMIZ2-E2F4, and ZMIZ2-DHX38 were obtained, which were enriched in DNA replication, cell cycle, and spliceosome, respectively, suggesting that these genes and corresponding proliferation-related pathways might contribute to TNBC development.

#### The AR regulatory pathway in TNBC samples

**Fig. 4:**
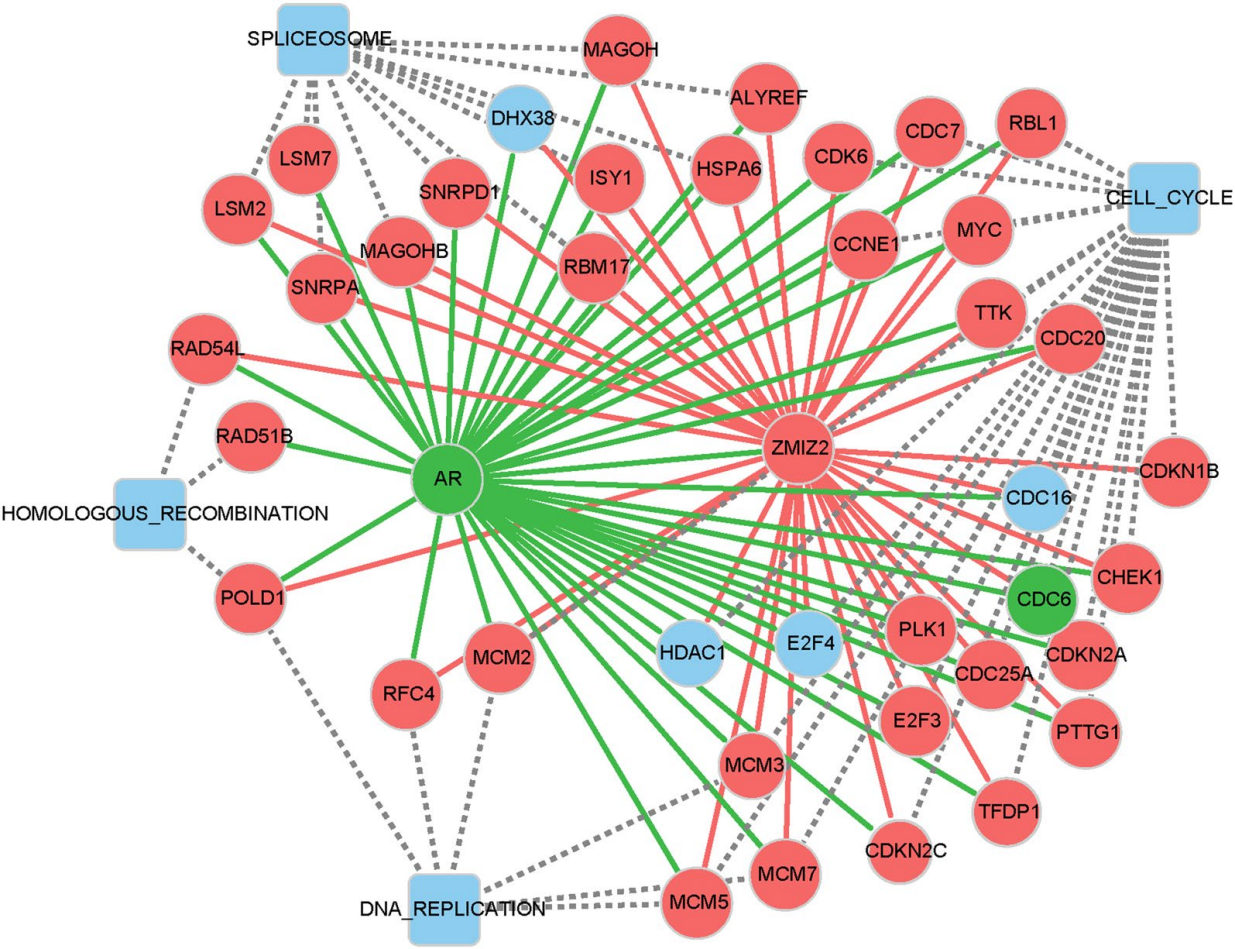
Regulatory mechanism networks for ZMIZ2-AR based on the pathways between the TNBC and normal samples. Red circle nodes represent upregulated genes, green circle nodes represent downregulated genes, and blue circle nodes represent non-differentially expressed genes. Square nodes represent pathways

In the abovementioned analysis, there were negative correlations between ZMIZ2 and AR in the TNBC samples, and consequently, we conducted GSEA of the AR regulatory pathway. The results of the GSEA revealed the positive and negative pathways (top 20 based on NES) regulated by AR in the TNBC samples (Additional file 2: Table S5). Venn analysis showed that 44 ZMIZ2-positive pathways were the same as the AR-negative pathways, and 13 ZMIZ2-negative pathways were the same as the AR-positive pathways (Additional file 1: Fig. S1C). These data confirmed the negative correlations between ZMIZ2 and AR. Based on the GSEA results, we selected four proliferation-related pathways regulated by ZMIZ2-AR in the TNBC samples, including the spliceosome, DNA replication, cell cycle, and homologous recombination. When the r > 0.3, 185 and 173 genes belonging to four pathways were found to be associated with ZMIZ2 and AR, respectively. In combination with the DE mRNAs between the TNBC and normal samples and prognostic genes, the regulatory mechanism network for ZMIZ2-AR was constructed, including 4 pathways and 41 gene nodes (Fig. 4). Notably, key ZMIZ2-regulatory genes, such as MCM3, E2F4, and DHX38 were also regulated by AR. Key relationships, such as AR-MCM3, AR-E2F4, and AR-DHX38 were identified.

### ZMIZ2 expression was up-regulated in different breast cancer subtypes and was associated with poor prognosis in clinical TNBC patients

#### Expression of ZMIZ2 in different breast cancer subtypes

**Fig. 5:**
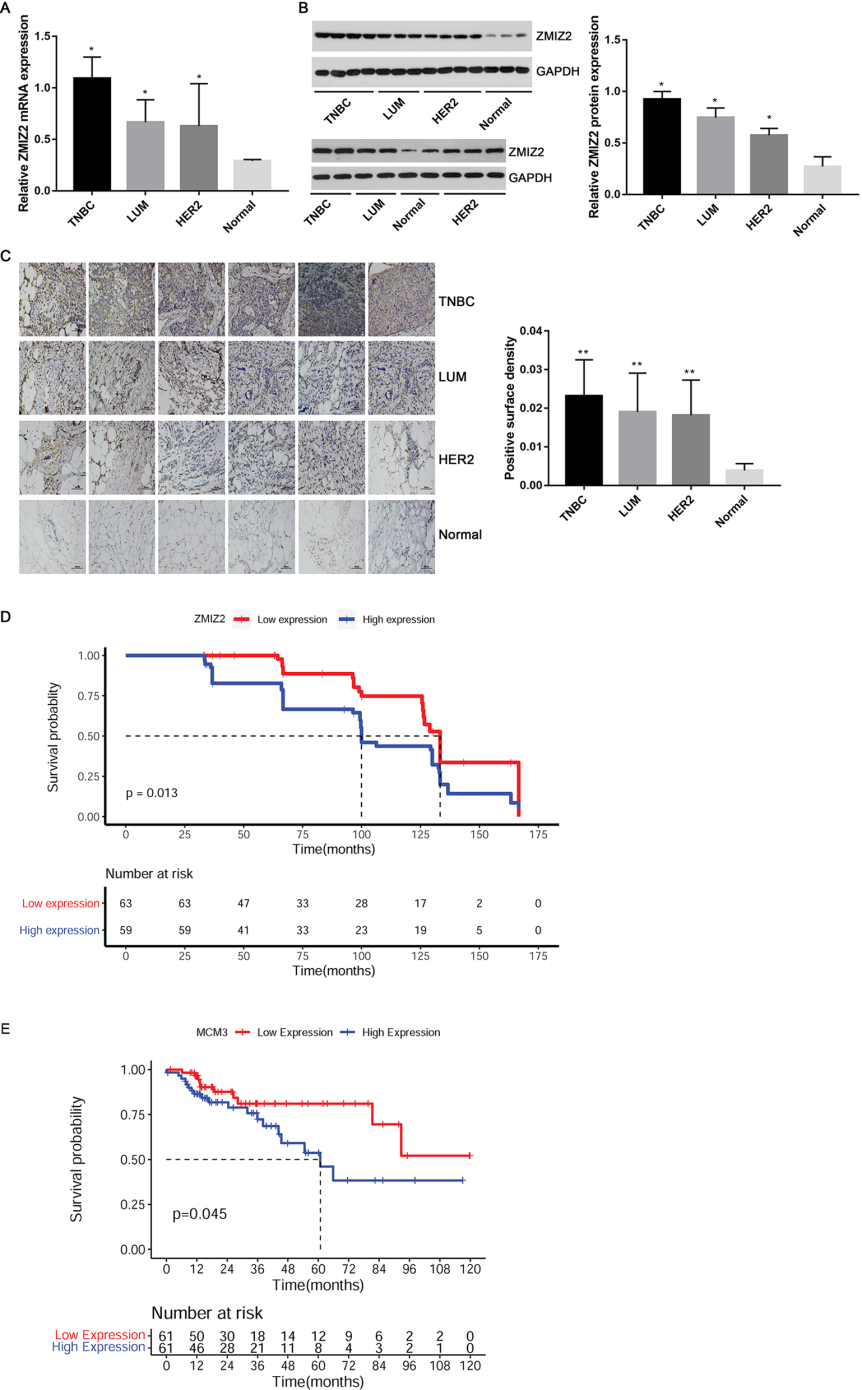
ZMIZ2 expression was upregulated in different breast cancer subtypes and associated with poor a prognosis in clinical TNBC patients. **A** qRT-PCR revealed the upregulation of ZMIZ2 mRNA expression in different breast cancer subtypes. **B** A Western blot assay showed the upregulation of ZMIZ2 protein expression in different breast cancer subtypes. **C** An immunohistochemical assay showed the upregulation of ZMIZ2 protein expression in different breast cancer subtypes. Magnification: 200 × ; bar scale: 100 μm. **D** Kaplan–Meier survival curves for high- and low-expression of ZMIZ2 in clinical patients with TNBC. E: Kaplan–Meier survival curves for high- and low-expression of MCM3 in clinical patients with TNBC. *P < 0.05 and **P < 0.01 when compared with the normal controls

The expression level of ZMIZ2 in the different breast cancer subtype tissue samples was quantified. qRT-PCR revealed that ZMIZ2 mRNA was significantly upregulated in cancer tissues in comparison with the controls (P < 0.05; Fig. 5A). Specifically, ZMIZ2 had the highest mRNA expression in the TNBC tissues, which was in accordance with the results of the aforementioned data analysis. Moreover, consistent results were obtained from western blot and immunohistochemistry: protein expression levels of ZMIZ2 were significantly increased in cancer tissues, especially the TNBC tissues, when compared with the control (P < 0.05, Fig. 5B, C).

#### The prognostic value of ZMIZ2 and MCM3 in TNBC

According to the median expression of ZMIZ2 or MCM3, TNBC samples were divided into high- and low-expression groups. High expression levels of ZMIZ2 or MCM3 were significantly correlated with a poor prognosis for clinical patients with TNBC (P = 0.013 and P = 0.045, respectively, Fig. 5D, E).

### In vitro validation of the ZMIZ2 expression in breast cancer cell lines and the oncogenic role of ZMIZ2 in TNBC cells

#### ZMIZ2 expression was increased in breast cancer cells

**Fig. 6:**
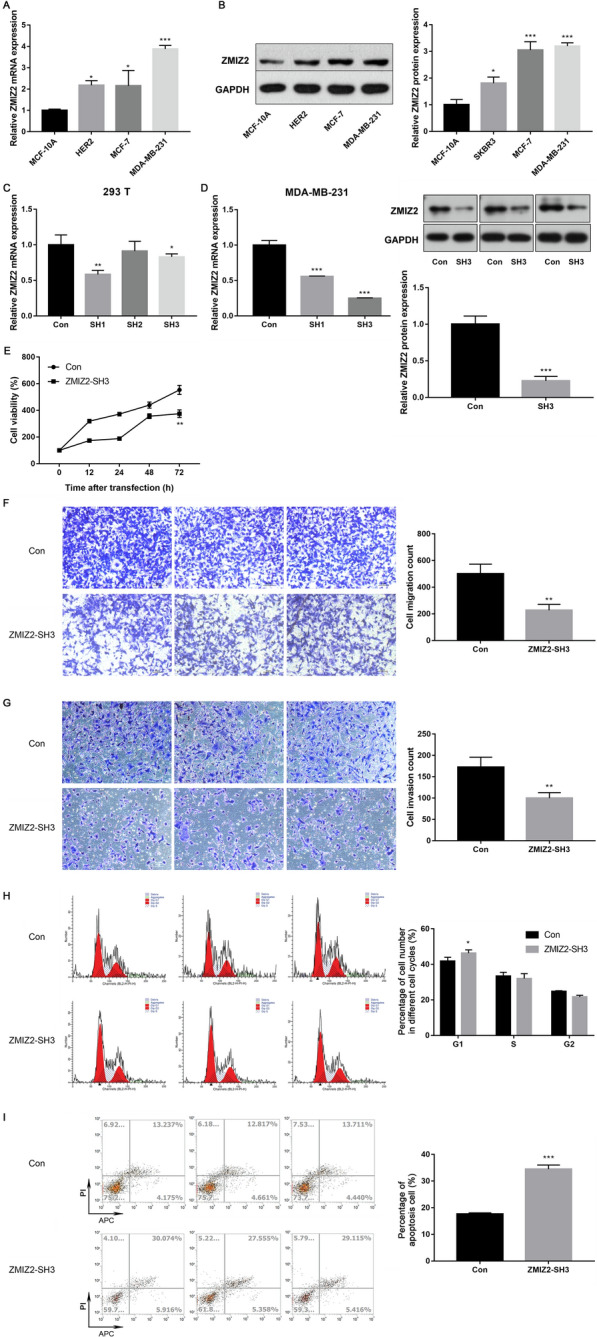
ZMIZ2 expression was upregulated in different breast cancer subtype cells, and the knockdown of ZMIZ2 inhibited the malignant behaviors of MDA-MB-231 cells. **A** qRT-PCR revealed the upregulation of ZMIZ2 mRNA expression in different breast cancer subtype cells. **B** Western blot assay showed the upregulation of ZMIZ2 protein expression in different breast cancer subtype cells. **C** ZMIZ2 was successfully knocked down in 293 T cells by transfection. **D** qRT-PCR and western blot assays indicated that ZMIZ2 was successfully knocked down in MDA-MB-231 cells by transfection. **E** CCK8 assay showed that the cell viability of MDA-MB-231 cells was significantly suppressed after the knockdown of ZMIZ2. **F**–**G** Transwell assays confirmed that the knockdown of ZMIZ2 significantly inhibited the migration and invasion of MDA-MB-231 cells. Magnification: 100 × ; bar scale: 50 μm. **H** Cell cycle analysis revealed that the knockdown of ZMIZ2 induced G1 phase arrest. **I** Flow cytometry showed that the knockdown of ZMIZ2 promoted MDA-MB-231 cell apoptosis. *P < 0.05, **P < 0.01, and ***P < 0.001 when compared with the corresponding controls

The expression levels of ZMIZ2 were assessed in the three breast cancer cell lines and the breast epithelial cells. As expected, the mRNA and protein expression levels of ZMIZ2 were upregulated in MDA-MB-231, MCF-7, and SKBR-3 cells when compared with the MCF-10A cells (P < 0.05, Fig. 6A, B). Specifically, the expression of ZMIZ2 was highest in the MDA-MB-231 cells.

**Fig. 7:**
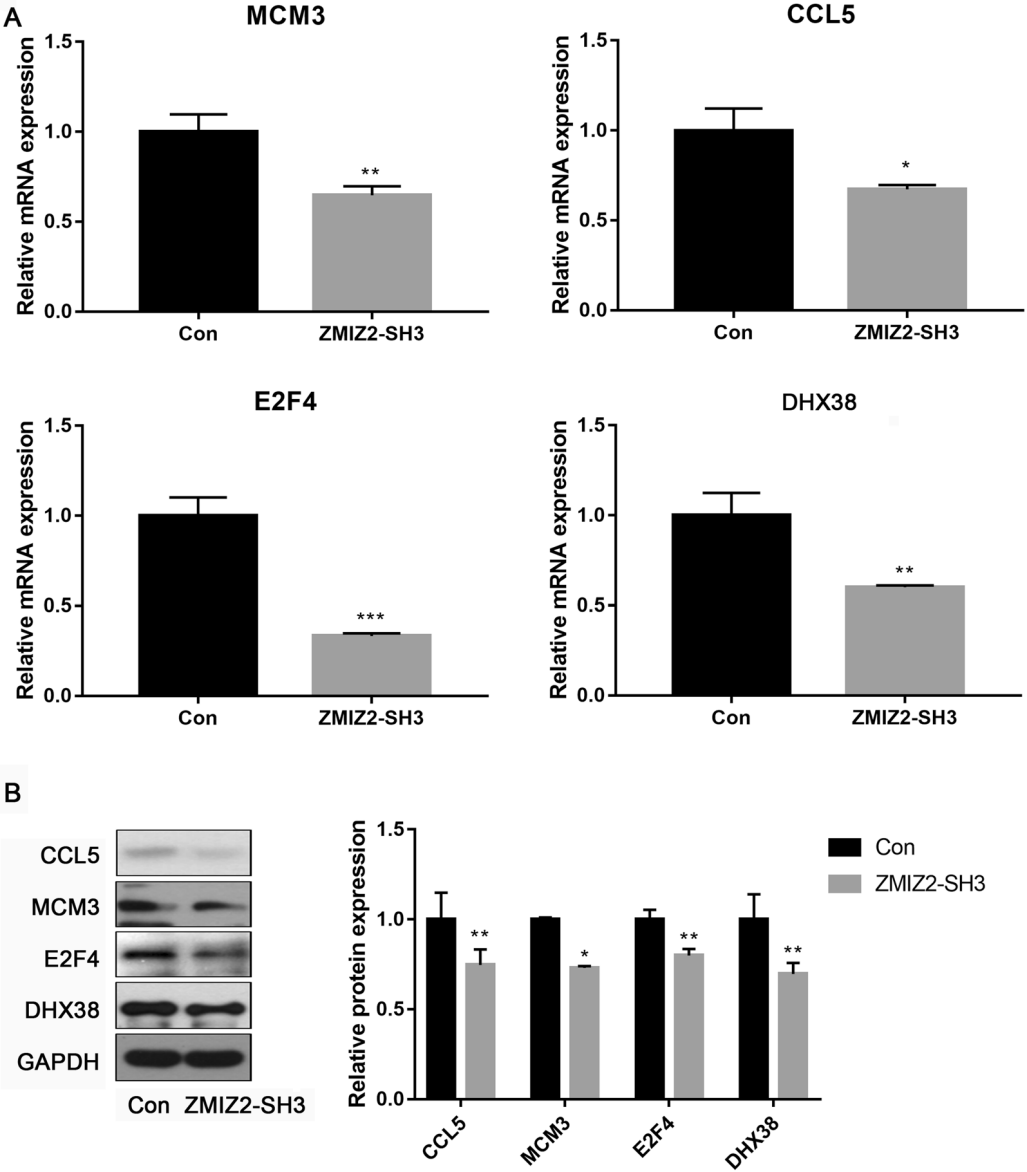
Expression of key targets of ZMIZ2 after the knockdown of ZMIZ2. **A** qRT-PCR revealed that the mRNA expression levels of CCL5, MCM3, E2F4, and DHX38 were decreased after the knockdown of ZMIZ2. **B** A Western blot assay showed that the protein expression levels of CCL5, MCM3, E2F4, and DHX38 were decreased after the knockdown of ZMIZ2. *P < 0.05, **P < 0.01, and ***P < 0.001 when compared with the controls

#### ZMIZ2 was successfully knocked down after transfection

To investigate the role of ZMIZ2 in TNBC, ZMIZ2 was knocked down by transfection with shRNAs targeting ZMIZ2. As revealed in Fig. 6C, ZMIZ2 was successfully knocked down in 293T cells by transfection with SH1 or SH3 (P < 0.05). The MDA-MB-231 cells were then further transfected with SH1 or SH3 to confirm their inhibitory effects on ZMIZ2 expression. Compared with the control, SH1 or SH3 transfection resulted in a significant decrease in ZMIZ2 mRNA expression (P < 0.05, Fig. 6D). Similarly, the results of western blot assay showed that ZMIZ2 protein expression was significantly decreased in the MDA-MB-231 cells transfected with ZMIZ2-SH3 (P < 0.05, Fig. 7B). These data confirmed that ZMIZ2 was successfully knocked down in the MDA-MB-231 cells after transfection. SH3 was then selected in subsequent experiments as it had a stronger inhibitory effect than SH1 in the MDA-MB-231 cells.

#### Knockdown of ZMIZ2 inhibited MDA-MB-231 cell proliferation, migration, and invasion, induced cell cycle arrest in G1 phase, and promoted cell apoptosis

To explore the function of ZMIZ2 in TNBC, the in vitro effects of the ZMIZ2 knockdown on MDA-MB-231 cell proliferation, migration, invasion, cell cycle, and apoptosis were investigated. The results of the CCK8 showed that the cell viability of the MDA-MB-231 cells was significantly suppressed after the knockdown of ZMIZ2 by transfection with SH3 (P < 0.01, Fig. 6E). The results of the Transwell assays confirmed that the knockdown of ZMIZ2 significantly inhibited the migration and invasion (P < 0.01, Fig. 6F, G). Moreover, cell cycle analysis revealed that MDA-MB-231 cells that were arrested in the G1 phase were dramatically increased in the ZMIZ2-SH3 group when compared to the control group (P < 0.01, Fig. 6H). Furthermore, the results of the flow cytometry for apoptosis detection showed that the percentage of apoptosis cells in the ZMIZ2-SH3 group was obviously higher than that in the control group (P < 0.001, Fig. 6I). Taken together, our results indicate that the knockdown of ZMIZ2 inhibited the malignant behaviors of TNBC cells, suggesting an oncogenic role for ZMIZ2 in TNBC.

#### Validation of the expression of key targets of ZMIZ2 in the MDA-MB-231 cells

Based on the above bioinformatics analysis, key targets of ZMIZ2, such as CCL5, MCM3, E2F4, and DHX38 were selected for validation, as they were found to be significantly correlated with ZMIZ2 and had prognostic value in patients with TNBC. CCL5, MCM3, E2F4, and DHX38 mRNA expression was significantly decreased in the ZMIZ2-SH3 group when compared to the control group (P < 0.05; Fig. 7A). Moreover, we quantified the protein expression levels of CCL5, MCM3, E2F4, and DHX38 via western blot assay. The results showed that, as expected, knockdown of ZMIZ2 resulted in significant decreases in the protein expression levels of CCL5, MCM3, E2F4, and DHX38 (P < 0.05, Fig. 7B). These data suggest that ZMIZ2 might contribute to TNBC development via interactions with these targets.

## Discussion

TNBC is an aggressive breast tumor subtype with a poor prognosis, that requires improved and more targeted treatment options [17, 18]. This is in-part because the molecular mechanisms underlying TNBC have not yet been completely elucidated. In the present study, the roles, and possible mechanisms of ZMIZ2 in TNBC were explored. Based on TCGA data, we found that ZMIZ2 was significantly upregulated in TNBC tissues and had significant negative correlations with AR in TNBC tissues. Following a series of bioinformatics analyses, crucial relationships, such as ZMIZ2-CCL5, ZMIZ2/AR-MCM3, ZMIZ2/AR-E2F4, and ZMIZ2/AR-DHX38 were identified. Moreover, based on the clinical samples, ZMIZ2 was confirmed to be upregulated in clinical breast cancer tissues and its high expression levels were correlated with a poor prognosis for TNBC patients. Furthermore, in vitro experiments revealed that ZMIZ2 expression was increased in breast cancer cells, and the knockdown of ZMIZ2 inhibited MDA-MB-231 cell proliferation, migration, and invasion, induced cell cycle arrest in G1 phase, and promoted cell apoptosis. Furthermore, the mRNA and protein expression of the key targets of ZMIZ2, including CCL5, MCM3, E2F4, and DHX38 were inhibited after its knockdown. To the best of our best knowledge, this is the first study to demonstrate the role and potential regulatory mechanisms of ZMIZ2 in TNBC.

ZMIZ2 is a PIAS-like protein that can modulate the activity of different TFs, such as p53, SMAD family members (Smads), and nuclear hormone receptors [19, 20]. p53 is a well-known tumor suppressor, the inactivation of which is implicated in the aggressiveness of TNBC as it promotes metastasis, and resistance to therapy [21]. Smads are key downstream targets of the activin-signaling pathway [22]. Activin and activin-signaling receptors have also been shown to play a pivotal role in maintaining control over cellular proliferation in breast cancer [23]. In addition, ZMIZ2 is identified as an AR-interacting protein that can augment AR-mediated transcription [24]. AR is reported to be expressed in 10–50% of TNBCs and may play a pivotal role in their carcinogenesis [9]. These results all indirectly indicate that ZMIZ2 has a critical role in TNBC. By combining data from TCGA, clinical tissue studies, and in vitro studies, this study has elucidated that ZMIZ2 is upregulated in TNBC tissues and cells. Moreover, for the first time, we have shown that knockdowns of ZMIZ2 inhibit MDA-MB-231 cell proliferation, migration, and invasion, induced cell cycle arrest in the G1 phase, and promoted cell apoptosis. These findings further reveal the oncogenic role of ZMIZ2 in TNBC and suggest its potential as a therapeutic target in the future. Furthermore, ZMIZ2 may regulate other genes through its PIAS activity or through transcriptional regulation. Although ZMIZ2 has been shown to augment AR-mediated transcription via its PIAS activity [24], the effects of ZMIZ2 on AR expression via transcriptional regulation were previously largely unknown. Our study revealed that ZMIZ2 expression was negatively correlated with AR expression in TNBC, using TCGA data. We thus speculated that AR expression and activity may be regulated by different mechanisms. Further experiments are required to confirm their relationships.

The interplay between individual cells in the tumor, the microenvironment, as well as the immune system contributes to cancer progression [25]. Some recent studies have revealed that when compared with other types of breast cancers, there are increased tumor-infiltrating lymphocytes in TNBC. Additionally, there are correlations between specific immune subsets and prognosis, but this requires more clinical investigation of their potential as immunotherapeutic agents in TNBC [26, 27]. In accordance with the reports above, our study showed that among the top 20 positive pathways regulated by ZMIZ2 in TNBC when compared to normal samples, 14 were immune system- and immune disease-related, which further indicated the critical role of the immune system in modulating the progression of TNBC. Based on these immune-related pathways and ZMIZ2-correlated genes, a ZMIZ2-regulated immune pathway-gene network was constructed. In this network, CCL5 was a prognostic gene, which was involved in the NOD-like receptor signaling pathway and toll-like receptor signaling pathway. It has been reported that the NOD-like receptor signaling pathway is active in many diseases, including inflammatory and autoimmune disorders, and cancer [28]. Yimam et al. [29] reported that the stimulation of NOD-like receptors could activate mitogen-activated protein kinases and nuclear factor-κB to drive the transcription of genes that were involved in innate and adaptive immune responses. A recent study has demonstrated that the upregulation of ubiquitin specific protease 21 promotes the tumorigenic capability of TNBC cells via the NOD-like receptor signaling pathway [30]. Toll-like receptors are primarily expressed in human immune and epithelial cells, and they function to trigger inflammatory responses by promoting the synthesis and release of inflammatory cytokines [31, 32]. Importantly, several toll-like receptors have been correlated with TNBC [33, 34]. Therefore, we speculated that ZMIZ2 may promote the progression of TNBC by regulating CCL5 to activate the NOD-like receptor signaling pathway and toll-like receptor signaling pathway. Notably, among the positive pathways regulated by ZMIZ2, the most common pathways in both the TNBC and HER2 samples were immune-related, such as the NOD like receptor signaling pathway. These data further suggest that both TNBC and HER2 subtypes may be closely associated with immunity.

Furthermore, among the specific positive pathways regulated by ZMIZ2 in TNBC, when compared with the HER2 samples, most were associated with proliferation, such as the spliceosome, DNA replication, and cell cycle pathways. Moreover, these proliferation-related pathways were regulated by AR in TNBC samples. It is well-known that the proliferation of neoplastic cells is an important feature during tumor growth. Thus, we speculated that ZMIZ2 and AR may be involved in TNBC development by regulating the key pathways related to proliferation. In addition, MCM proteins are a group of helicases, which play a fundamental role in the replication of eukaryotic DNA [35]. The MCM3 protein is present in quiescent or differentiated cells at lower intracellular levels. Importantly, MCM3 is overexpressed in some human cancers, including breast cancers [36, 37]. The phosphorylation of MCM3 can negatively regulate DNA replication and checkpoint activation [38]. Moreover, overexpression of lncRNA CARMN promotes the prognosis and chemosensitivity of TNBC through the inhibition of DNA replication [39], suggesting a potential role of DNA replication in TNBC. In this study, ZMIZ2, AR, and MCM3 were enriched in DNA replication, and ZMIZ2/AR-MCM3 relationships were obtained. Therefore, we speculated that ZMIZ2-MCM3 and AR-MCM3 may be key relationships pertaining to DNA replication. E2F4 is a key regulator of cell cycle that is widely associated with tumorigenesis and cancer severity [40, 41]. Cell cycle genes have been revealed to be evolutionarily conserved targets of E2F4 [42], and E2F4 expression is required for cell cycle progression of colorectal cancer cells [43]. Herein, ZMIZ2/AR-E2F4 relationships were obtained, and these three genes were enriched in cell cycle, suggesting that ZMIZ2-E2F4 and AR-E2F4 were crucial relationships related to cell cycle. DHX38 is a slicing factor that is involved in the etiology of early-onset retinitis pigmentosa [44, 45]. Fraile et al. has demonstrated that DHX38 encodes an USP39-interacting splicing factor, whose depletion reduces the viability of KRAS-active cells, and thus, affects KRAS-driven cancers [46]. The crucial relationships ZMIZ2/AR-DHX38 were obtained, and these three genes were enriched in spliceosome. Therefore, we speculate that ZMIZ2-DHX38 and AR-DHX38 may affect the spliceosome to contribute to the proliferation of TNBC cells. Given the negative correlation between ZMIZ2 expression and AR expression, it is not clear whether they have opposite effects when they modulate the same pathway. Further in-depth investigation of the underlying mechanism is warranted to elucidate their roles in these pathways.

There are some limitations to our study. First, the negative correlation between ZMIZ2 expression and AR expression was not validated in the clinical patient samples. Second, key relationships such as ZMIZ2/AR-MCM3, ZMIZ2/AR-E2F4, and ZMIZ2/AR-DHX38 were not confirmed via experimental validation. Finally, whether ZIMZ2 was modulated at different stages of breast cancer was not determined due to lack of public or clinical data. Further studies with large samples and more experiments are required to verify our observations.

## Conclusions

In conclusion, this study has demonstrated for the first time that ZMIZ2 is upregulated in TNBC, and that this may be associated with a poor prognosis for TNBC patients. Knockdowns of ZMIZ2 may inhibit TNBC cell proliferation, migration, and invasion, induce cell cycle arrest in the G1 phase, and promote cell apoptosis. Further analysis suggests that ZMIZ2 may promote the progression of TNBC by promoting CCL5, which affects several immune-related pathways, such as the NOD-like receptor signaling pathway and the toll-like receptor signaling pathway. Additionally, ZMIZ2 is negatively correlated with AR, and ZMIZ2 and AR may promote the proliferation of TNBC cells by regulating MCM3-DNA replication, the E2F4-cell cycle, and the DHX38-spliceosome. ZMIZ2 may thus serve as a promising biomarker or target for TNBC in the future.

## Supplementary Information

**Additional file 1: Fig. S1.** Venn analysis showed the key pathways regulated by ZMIZ2 between the TNBC and normal samples (A), and key pathways regulated by ZMIZ2 between the TNBC and HER2 samples (B), as well the key pathways regulated by ZMIZ2 and AR (C).

**Additional file 2: Table S1.** The sequences of the short hairpin RNAs (shRNAs) targeting ZMIZ2. **Table S2.** The positive and negative pathways (top 20 based on NES) regulated by ZMIZ2 in the TNBC samples. **Table S3.** The positive and negative pathways (top 20 based on NES) regulated by ZMIZ2 in the normal samples. **Table S4.** The positive and negative pathways (top 20 based on NES) regulated by ZMIZ2 in the HER2 samples. **Table S4.** The positive and negative pathways (top 20 based on NES) regulated by ZMIZ2 in the HER2 samples.

## Data Availability

All data generated or analyzed during this study are included in this published article.

## Abbreviations

TNBC: Triple-receptor negative breast cancer
TCGA: The Cancer Genome Atlas
ER: Estrogen receptor
PR: Progesterone receptor
HER2: Human epidermal growth factor receptor 2
ZMIZ2: Zinc finger MIZ-type containing 2
PIAS: Protein inhibitor of activated STAT
AR: Androgen receptor
FC: Fold change
TF: Transcription factor
GSEA: Gene set enrichment analysis
qRT-PCR: Real-time quantitative reverse transcription-PCR
PVDF: Polyvinylidene fluoride
FBS: Fetal bovine serum
shRNA: Short hairpin RNA
SD: Standard deviation
