# Supplementary figures and images for: ZMIZ2 promotes the development of triple-receptor negative breast cancer
Source: Cancer Cell Int. 2022 Jan 31;22:52. doi: 10.1186/s12935-021-02393-x (PMC8802436; doi:10.1186/s12935-021-02393-x)

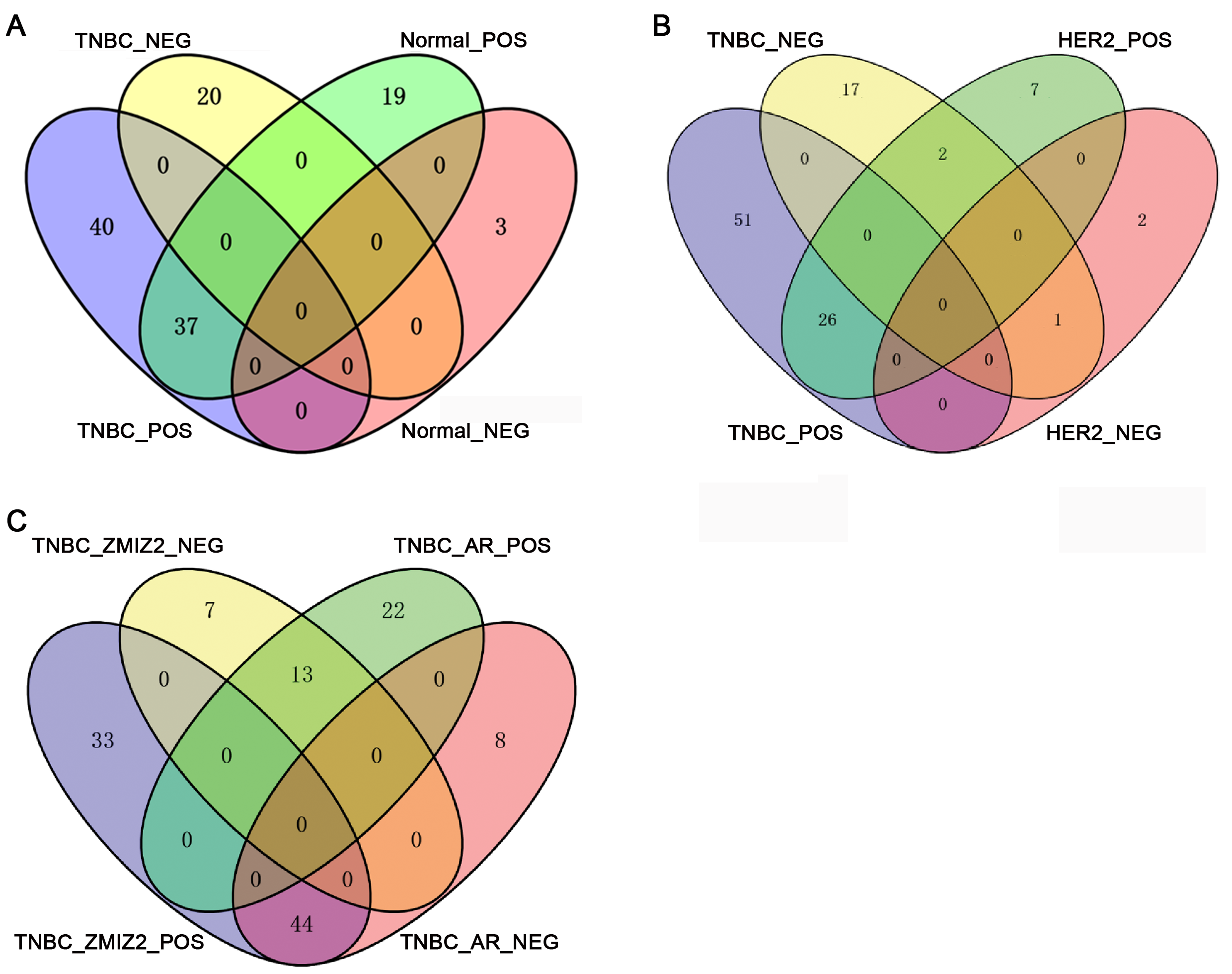

Supplement: Supplementary file 1 — Additional file 1: Fig. S1. Venn analysis showed the key pathways regulated by ZMIZ2 between the TNBC and normal samples (A), and key pathways regulated by ZMIZ2 between the TNBC and HER2 samples (B), as well the key pathways regulated by ZMIZ2 and AR (C). [file 12935_2021_2393_MOESM1_ESM.tif]
