# Supplementary material for: ZMIZ2 promotes the development of triple-receptor negative breast cancer
Source: Cancer Cell Int. 2022 Jan 31;22:52. doi: 10.1186/s12935-021-02393-x (PMC8802436; doi:10.1186/s12935-021-02393-x)
Supplement: Supplementary file 2 — Additional file 2: Table S1. The sequences of the short hairpin RNAs (shRNAs) targeting ZMIZ2. Table S2. The positive and negative pathways (top 20 based on NES) regulated by ZMIZ2 in the TNBC samples. Table S3. The positive and negative pathways (top 20 based on NES) regulated by ZMIZ2 in the normal samples. Table S4. The positive and negative pathways (top 20 based on NES) regulated by ZMIZ2 in the HER2 samples. Table S4. The positive and negative pathways (top 20 based on NES) regulated by ZMIZ2 in the HER2 samples. [file 12935_2021_2393_MOESM2_ESM.docx]

**Table S1.** The sequences of short hairpin RNA (shRNA) targeting *ZMIZ2*

| shRNAs | Forward | Reverse |
| --- | --- | --- |
| SH1 | TGCTGTTGACAGTGAGCGCGCTGGGCATCCTGATTTACATTAGTGAAGCCACAGATGTAATGTAAATCAGGATGCCCAGCATGCCTACTGCCTCGGA | TCCGAGGCAGTAGGCATGCTGGGCATCCTGATTTACATTACATCTGTGGCTTCACTAATGTAAATCAGGATGCCCAGCGCGCTCACTGTCAACAGCA |
| SH2 | TGCTGTTGACAGTGAGCGACGCCTGCCTCCCAAGCCAGATTAGTGAAGCCACAGATGTAATCTGGCTTGGGAGGCAGGCGGTGCCTACTGCCTCGGA | TCCGAGGCAGTAGGCACCGCCTGCCTCCCAAGCCAGATTACATCTGTGGCTTCACTAATCTGGCTTGGGAGGCAGGCGTCGCTCACTGTCAACAGCA |
| SH3 | TGCTGTTGACAGTGAGCGACCAGCTGCGAGACTCAGTCTATAGTGAAGCCACAGATGTATAGACTGAGTCTCGCAGCTGGATGCCTACTGCCTCGGA | TCCGAGGCAGTAGGCATCCAGCTGCGAGACTCAGTCTATACATCTGTGGCTTCACTATAGACTGAGTCTCGCAGCTGGTCGCTCACTGTCAACAGCA |

**Table S2.** The positive and negative pathways (top 20 based on NES) regulated by *ZMIZ2* in TNBC samples

| Name | SIZE | NES | NOM p-value | FDR q-value |
| --- | --- | --- | --- | --- |
| Positive pathways |  |  |  |  |
| KEGG_T_CELL_RECEPTOR_SIGNALING_PATHWAY | 107 | 2.525814 | 0 | 0 |
| KEGG_ALLOGRAFT_REJECTION | 35 | 2.5076501 | 0 | 0 |
| KEGG_LEISHMANIA_INFECTION | 69 | 2.4929893 | 0 | 0 |
| KEGG_SPLICEOSOME | 126 | 2.4922001 | 0 | 0 |
| KEGG_DNA_REPLICATION | 36 | 2.4673064 | 0 | 0 |
| KEGG_CELL_CYCLE | 124 | 2.4557598 | 0 | 0 |
| KEGG_NOD_LIKE_RECEPTOR_SIGNALING_PATHWAY | 61 | 2.416907 | 0 | 0 |
| KEGG_GRAFT_VERSUS_HOST_DISEASE | 37 | 2.3912017 | 0 | 0 |
| KEGG_ANTIGEN_PROCESSING_AND_PRESENTATION | 81 | 2.3886569 | 0 | 0 |
| KEGG_PRIMARY_IMMUNODEFICIENCY | 35 | 2.3744943 | 0 | 0 |
| KEGG_HOMOLOGOUS_RECOMBINATION | 26 | 2.3527417 | 0 | 0 |
| KEGG_NATURAL_KILLER_CELL_MEDIATED_CYTOTOXICITY | 132 | 2.350802 | 0 | 0 |
| KEGG_TYPE_I_DIABETES_MELLITUS | 41 | 2.3472748 | 0 | 0 |
| KEGG_CHEMOKINE_SIGNALING_PATHWAY | 185 | 2.3357153 | 0 | 0 |
| KEGG_SYSTEMIC_LUPUS_ERYTHEMATOSUS | 55 | 2.3204288 | 0 | 0 |
| KEGG_B_CELL_RECEPTOR_SIGNALING_PATHWAY | 75 | 2.2692728 | 0 | 0 |
| KEGG_INTESTINAL_IMMUNE_NETWORK_FOR_IGA_PRODUCTION | 46 | 2.247273 | 0 | 0 |
| KEGG_AUTOIMMUNE_THYROID_DISEASE | 50 | 2.2273605 | 0 | 0 |
| KEGG_TOLL_LIKE_RECEPTOR_SIGNALING_PATHWAY | 101 | 2.2212996 | 0 | 0 |
| KEGG_MISMATCH_REPAIR | 23 | 2.1850102 | 0 | 0 |
| Negative pathways |  |  |  |  |
| KEGG_PARKINSONS_DISEASE | 113 | -2.4715288 | 0 | 0 |
| KEGG_OXIDATIVE_PHOSPHORYLATION | 115 | -2.4204533 | 0 | 0 |
| KEGG_STEROID_HORMONE_BIOSYNTHESIS | 55 | -2.1218565 | 0 | 0.004424112 |
| KEGG_RIBOSOME | 88 | -2.1128879 | 0 | 0.003318084 |
| KEGG_ALZHEIMERS_DISEASE | 152 | -2.026984 | 0 | 0.004052033 |
| KEGG_TYROSINE_METABOLISM | 40 | -2.0135598 | 0 | 0.004308929 |
| KEGG_DRUG_METABOLISM_CYTOCHROME_P450 | 71 | -1.9127986 | 0 | 0.007916221 |
| KEGG_CARDIAC_MUSCLE_CONTRACTION | 78 | -1.8343853 | 0 | 0.015389929 |
| KEGG_ECM_RECEPTOR_INTERACTION | 84 | -1.805953 | 0 | 0.017326966 |
| KEGG_RETINOL_METABOLISM | 64 | -1.7834733 | 0 | 0.017644502 |
| KEGG_METABOLISM_OF_XENOBIOTICS_BY_CYTOCHROME_P450 | 69 | -1.7705309 | 0 | 0.018581288 |
| KEGG_ABC_TRANSPORTERS | 44 | -1.7344285 | 0.00591716 | 0.022092525 |
| KEGG_HUNTINGTONS_DISEASE | 167 | -1.6711887 | 0 | 0.03350761 |
| KEGG_PENTOSE_AND_GLUCURONATE_INTERCONVERSIONS | 28 | -1.6580747 | 0.01 | 0.034172993 |
| KEGG_PEROXISOME | 78 | -1.6388022 | 0 | 0.03566065 |
| KEGG_PROPANOATE_METABOLISM | 32 | -1.6366117 | 0.010752688 | 0.034066107 |
| KEGG_VALINE_LEUCINE_AND_ISOLEUCINE_DEGRADATION | 43 | -1.5873517 | 0.013333334 | 0.044672314 |
| KEGG_GLYCOSYLPHOSPHATIDYLINOSITOL_GPI_ANCHOR_BIOSYNTHESIS | 25 | -1.5845608 | 0.030837005 | 0.04310424 |
| KEGG_PHENYLALANINE_METABOLISM | 17 | -1.5397834 | 0.041198503 | 0.055521354 |
| KEGG_CALCIUM_SIGNALING_PATHWAY | 177 | -1.5258262 | 0 | 0.05817793 |

**Table S3.** The positive and negative pathways (top 20 based on NES) regulated by *ZMIZ2* in normal samples

| Name | Size | NES | NOM p-value | FDR q-value |
| --- | --- | --- | --- | --- |
| Positive pathways |  |  |  |  |
| KEGG_PYRIMIDINE_METABOLISM | 98 | 1.970865 | 0.001742 | 0.271954 |
| KEGG_PURINE_METABOLISM | 154 | 1.926745 | 0.001647 | 0.236761 |
| KEGG_SELENOAMINO_ACID_METABOLISM | 24 | 1.924332 | 0.001751 | 0.16203 |
| KEGG_P53_SIGNALING_PATHWAY | 66 | 1.895645 | 0.012389 | 0.165156 |
| KEGG_INOSITOL_PHOSPHATE_METABOLISM | 54 | 1.877559 | 0.001742 | 0.159858 |
| KEGG_CELL_CYCLE | 124 | 1.870731 | 0.003546 | 0.144593 |
| KEGG_GLYCOSPHINGOLIPID_BIOSYNTHESIS_LACTO_AND_NEOLACTO_SERIES | 26 | 1.86914 | 0.003436 | 0.127014 |
| KEGG_NUCLEOTIDE_EXCISION_REPAIR | 44 | 1.860304 | 0.006838 | 0.122944 |
| KEGG_SMALL_CELL_LUNG_CANCER | 84 | 1.855552 | 0 | 0.113911 |
| KEGG_SPLICEOSOME | 126 | 1.850732 | 0 | 0.107277 |
| KEGG_BASAL_CELL_CARCINOMA | 55 | 1.850691 | 0.007168 | 0.097525 |
| KEGG_PATHWAYS_IN_CANCER | 322 | 1.845615 | 0.001727 | 0.093376 |
| KEGG_ARRHYTHMOGENIC_RIGHT_VENTRICULAR_CARDIOMYOPATHY_ARVC | 74 | 1.824757 | 0.005076 | 0.10325 |
| KEGG_HOMOLOGOUS_RECOMBINATION | 26 | 1.82307 | 0.003515 | 0.097637 |
| KEGG_GLYCEROPHOSPHOLIPID_METABOLISM | 72 | 1.818542 | 0.006689 | 0.096019 |
| KEGG_PHOSPHATIDYLINOSITOL_SIGNALING_SYSTEM | 76 | 1.816262 | 0.003584 | 0.092278 |
| KEGG_EPITHELIAL_CELL_SIGNALING_IN_HELICOBACTER_PYLORI_INFECTION | 68 | 1.816193 | 0.007042 | 0.08685 |
| KEGG_UBIQUITIN_MEDIATED_PROTEOLYSIS | 130 | 1.801646 | 0.015025 | 0.094306 |
| KEGG_HEDGEHOG_SIGNALING_PATHWAY | 56 | 1.795103 | 0.001773 | 0.093924 |
| KEGG_NOTCH_SIGNALING_PATHWAY | 47 | 1.788533 | 0.014035 | 0.09498 |
| Negative pathways |  |  |  |  |
| KEGG_CITRATE_CYCLE_TCA_CYCLE | 31 | -1.67548 | 0.027088 | 0.42372 |
| KEGG_FATTY_ACID_METABOLISM | 42 | -1.61832 | 0.037209 | 0.224252 |
| KEGG_PYRUVATE_METABOLISM | 40 | -1.58807 | 0.042755 | 0.166915 |

**Table S4.** The positive and negative pathways (top 20 based on NES) regulated by *ZMIZ2* in HER2 samples

| NAME | SIZE | NES | NOM p-val | FDR q-val |
| --- | --- | --- | --- | --- |
| Positive pathways |  |  |  |  |
| KEGG_GRAFT_VERSUS_HOST_DISEASE | 37 | 2.1653404 | 0 | 0 |
| KEGG_VIRAL_MYOCARDITIS | 68 | 2.118592 | 0 | 0 |
| KEGG_PRIMARY_IMMUNODEFICIENCY | 35 | 2.1108236 | 0 | 0 |
| KEGG_ALLOGRAFT_REJECTION | 35 | 2.0135906 | 0 | 0.001205535 |
| KEGG_SYSTEMIC_LUPUS_ERYTHEMATOSUS | 55 | 2.002434 | 0 | 0.001158614 |
| KEGG_LEISHMANIA_INFECTION | 69 | 1.9971036 | 0 | 0.001125474 |
| KEGG_AUTOIMMUNE_THYROID_DISEASE | 50 | 1.9931511 | 0 | 9.65E-04 |
| KEGG_TYPE_I_DIABETES_MELLITUS | 41 | 1.9632291 | 0 | 0.001690161 |
| KEGG_INTESTINAL_IMMUNE_NETWORK_FOR_IGA_PRODUCTION | 46 | 1.9487525 | 0 | 0.001824791 |
| KEGG_HEMATOPOIETIC_CELL_LINEAGE | 85 | 1.8764654 | 0 | 0.006783569 |
| KEGG_ASTHMA | 28 | 1.8348497 | 0.002166847 | 0.012342091 |
| KEGG_CHEMOKINE_SIGNALING_PATHWAY | 185 | 1.8230604 | 0 | 0.014209443 |
| KEGG_ECM_RECEPTOR_INTERACTION | 84 | 1.8073884 | 0 | 0.016463133 |
| KEGG_CELL_ADHESION_MOLECULES_CAMS | 128 | 1.7748713 | 0 | 0.021946246 |
| KEGG_CYTOKINE_CYTOKINE_RECEPTOR_INTERACTION | 262 | 1.7286333 | 0 | 0.033788532 |
| KEGG_CYTOSOLIC_DNA_SENSING_PATHWAY | 53 | 1.7184823 | 0.00203252 | 0.035680067 |
| KEGG_B_CELL_RECEPTOR_SIGNALING_PATHWAY | 75 | 1.7046919 | 0 | 0.039449632 |
| KEGG_DILATED_CARDIOMYOPATHY | 90 | 1.6784155 | 0 | 0.049537543 |
| KEGG_FOCAL_ADHESION | 197 | 1.67248 | 0 | 0.04948833 |
| Negative pathways |  |  |  |  |
| KEGG_RIBOSOME | 113 | -2.5462403 | 0 | 0 |
| KEGG_PROTEIN_EXPORT | 88 | -2.0162363 | 0 | 0.00144589 |
| KEGG_N_GLYCAN_BIOSYNTHESIS | 23 | -1.669084 | 0 | 0.032369856 |
| KEGG_OXIDATIVE_PHOSPHORYLATION | 46 | -1.0632244 | 0 | 0.38917902 |

**Table S5.** The positive and negative pathways (top 20 based on NES) regulated by *AR* in TNBC samples

| NAME | SIZE | NES | NOM p-val | FDR q-val |
| --- | --- | --- | --- | --- |
| Positive pathways |  |  |  |  |
| KEGG_TYROSINE_METABOLISM | 40 | 2.3393152 | 0 | 0 |
| KEGG_STEROID_HORMONE_BIOSYNTHESIS | 55 | 2.2820768 | 0 | 0 |
| KEGG_PEROXISOME | 78 | 2.1625216 | 0 | 2.17E-04 |
| KEGG_DRUG_METABOLISM_CYTOCHROME_P450 | 71 | 2.1602848 | 0 | 1.62E-04 |
| KEGG_RETINOL_METABOLISM | 64 | 2.1510394 | 0 | 1.30E-04 |
| KEGG_COMPLEMENT_AND_COAGULATION_CASCADES | 69 | 2.1498346 | 0 | 1.08E-04 |
| KEGG_PENTOSE_AND_GLUCURONATE_INTERCONVERSIONS | 28 | 2.1424809 | 0 | 1.85E-04 |
| KEGG_PORPHYRIN_AND_CHLOROPHYLL_METABOLISM | 40 | 2.1238055 | 0 | 2.42E-04 |
| KEGG_ASCORBATE_AND_ALDARATE_METABOLISM | 25 | 2.0319932 | 0.001236094 | 4.30E-04 |
| KEGG_ABC_TRANSPORTERS | 44 | 2.0266852 | 0 | 3.87E-04 |
| KEGG_METABOLISM_OF_XENOBIOTICS_BY_CYTOCHROME_P450 | 69 | 2.0196939 | 0 | 4.71E-04 |
| KEGG_SPHINGOLIPID_METABOLISM | 36 | 1.9724946 | 0 | 0.001309132 |
| KEGG_VALINE_LEUCINE_AND_ISOLEUCINE_DEGRADATION | 43 | 1.956437 | 0 | 0.001611499 |
| KEGG_PHENYLALANINE_METABOLISM | 17 | 1.9533987 | 0 | 0.001543095 |
| KEGG_RENIN_ANGIOTENSIN_SYSTEM | 17 | 1.852833 | 0.00409836 | 0.005948224 |
| KEGG_PPAR_SIGNALING_PATHWAY | 69 | 1.840868 | 0 | 0.006433392 |
| KEGG_ARACHIDONIC_ACID_METABOLISM | 58 | 1.8071548 | 0.002247191 | 0.008969553 |
| KEGG_DRUG_METABOLISM_OTHER_ENZYMES | 51 | 1.8015176 | 0.002275313 | 0.008986669 |
| KEGG_OTHER_GLYCAN_DEGRADATION | 16 | 1.7848073 | 0.009421265 | 0.010412874 |
| KEGG_STARCH_AND_SUCROSE_METABOLISM | 52 | 1.7696159 | 0.002285714 | 0.011835807 |
| Negative pathways |  |  |  |  |
| KEGG_CELL_CYCLE | 124 | -3.6650248 | 0 | 0 |
| KEGG_SPLICEOSOME | 126 | -3.1282296 | 0 | 0 |
| KEGG_DNA_REPLICATION | 36 | -3.077139 | 0 | 0 |
| KEGG_GLYCOSPHINGOLIPID_BIOSYNTHESIS_LACTO_AND_NEOLACTO_SERIES | 26 | -2.5892463 | 0 | 0 |
| KEGG_HOMOLOGOUS_RECOMBINATION | 26 | -2.4274392 | 0 | 0 |
| KEGG_PROTEASOME | 45 | -2.3710291 | 0 | 4.52E-04 |
| KEGG_RNA_POLYMERASE | 29 | -2.3620195 | 0 | 3.88E-04 |
| KEGG_PYRIMIDINE_METABOLISM | 98 | -2.3240952 | 0 | 3.39E-04 |
| KEGG_RNA_DEGRADATION | 56 | -2.3150008 | 0 | 3.02E-04 |
| KEGG_ANTIGEN_PROCESSING_AND_PRESENTATION | 81 | -2.2153504 | 0 | 5.64E-04 |
| KEGG_MISMATCH_REPAIR | 23 | -2.1578815 | 0 | 0.001946066 |
| KEGG_DORSO_VENTRAL_AXIS_FORMATION | 24 | -2.1576502 | 0.004901961 | 0.001783894 |
| KEGG_NATURAL_KILLER_CELL_MEDIATED_CYTOTOXICITY | 132 | -2.0606391 | 0 | 0.002635028 |
| KEGG_NUCLEOTIDE_EXCISION_REPAIR | 44 | -2.0573084 | 0 | 0.002682836 |
| KEGG_NON_SMALL_CELL_LUNG_CANCER | 54 | -2.0198777 | 0 | 0.003155409 |
| KEGG_OOCYTE_MEIOSIS | 111 | -2.0147254 | 0 | 0.003901685 |
| KEGG_BASAL_TRANSCRIPTION_FACTORS | 35 | -1.9831665 | 0 | 0.004862678 |
| KEGG_PROGESTERONE_MEDIATED_OOCYTE_MATURATION | 85 | -1.9759008 | 0 | 0.004995986 |
| KEGG_GRAFT_VERSUS_HOST_DISEASE | 37 | -1.9693829 | 0 | 0.004998303 |
| KEGG_NOD_LIKE_RECEPTOR_SIGNALING_PATHWAY | 61 | -1.9664568 | 0 | 0.005121114 |
